# PEG-modified gadolinium nanoparticles as contrast agents for in vivo micro-CT

**DOI:** 10.1038/s41598-021-95716-x

**Published:** 2021-08-16

**Authors:** Charmainne Cruje, P. Joy Dunmore-Buyze, Eric Grolman, David W. Holdsworth, Elizabeth R. Gillies, Maria Drangova

**Affiliations:** 1 Robarts Research Institute, Western University, London, ON N6A 5B7 Canada; 2 Department of Medical Biophysics, Western University, London, ON N6A 5B7 Canada; 3 School of Biomedical Engineering, Western University, London, ON N6A 5B7 Canada; 4 Department of Chemistry, Western University, London, ON N6A 5B7 Canada; 5 Department of Chemical and Biochemical Engineering, Western University, London, ON N6A 5B7 Canada

**Keywords:** Preclinical research, Imaging techniques and agents, Biomedical engineering, Cardiovascular biology

## Abstract

Vascular research is largely performed in rodents with the goal of developing treatments for human disease. Micro-computed tomography (micro-CT) provides non-destructive three-dimensional imaging that can be used to study the vasculature of rodents. However, to distinguish vasculature from other soft tissues, long-circulating contrast agents are required. In this study, we demonstrated that poly(ethylene glycol) (PEG)-coated gadolinium nanoparticles can be used as a vascular contrast agent in micro-CT. The coated particles could be lyophilized and then redispersed in an aqueous solution to achieve 100 mg/mL of gadolinium. After an intravenous injection of the contrast agent into mice, micro-CT scans showed blood pool contrast enhancements of at least 200 HU for 30 min. Imaging and quantitative analysis of gadolinium in tissues showed the presence of contrast agent in clearance organs including the liver and spleen and very low amounts in other organs. In vitro cell culture experiments, subcutaneous injections, and analysis of mouse body weight suggested that the agents exhibited low toxicity. Histological analysis of tissues 5 days after injection of the contrast agent showed cytotoxicity in the spleen, but no abnormalities were observed in the liver, lungs, kidneys, and bladder.

## Introduction

Micro-computed tomography (micro-CT) provides a quantitative, non-destructive, fast and cost-effective means of studying vascular disease in mouse models^1–9^. In live mice, micro-CT can provide imaging resolution down to tens of micrometers within tens of minutes. Because CT contrast is derived from the density-dependent attenuation of x rays, soft tissues (which have similar densities) provide little differential contrast. Hence, x-ray attenuating contrast agents are injected intravenously to distinguish the vasculature from surrounding soft tissues during a micro-CT scan, enabling visualization and quantitative tracking of blood vessels, including during studies of novel therapies for re-vascularization^10–12^. For optimal utility in in vivo imaging, contrast agents must have a high initial concentration of a highly attenuating contrast element, circulate in the blood pool during the course of the micro-CT scan, and be cleared from the body after imaging to enable longitudinal studies with repeated injections. Clinically used contrast agents comprise small molecules that are cleared renally within seconds of intravenous administration; while these contrast agents meet the requirements of human imaging where scan times are short, in vivo micro-CT protocols require circulation times in the order of tens of minutes^13–17^.

Advances in nanotechnology and polymer science have enabled the development of commercially available agents that can evade immediate clearance from the blood-pool and circulate for prolonged periods in mice^16,18^. These agents are composed of nanoparticles with diameters greater than 10 nm, thus avoiding clearance via the kidneys^5,19–22^. To further evade clearance by the reticuloendothelial system (RES), carrier polymers that act as shields from the in vivo milieu are used to coat the nanoparticles. This “core–shell” design can also be used to deliver a high loading of contrast material in the core, making the design important for micro-CT contrast agents, where contrast-agent concentrations of at least 100 mg/mL are typically required. Poly(ethylene glycol) (PEG) has been widely utilized to coat nanoparticles to achieve long circulation times^23,24^ because of its stealthy properties with respect to the RES, high water solubility, low cytotoxicity, availability in different lengths, and a terminal group can be modified into functional groups to coat nanoparticles^25,26^. For example, phospholipid-terminated PEG is used in Fenestra VC, which encapsulates 50 mg/mL of iodine within lipid emulsions^8,20,27,28^, while 15 nm gold nanoparticles are coated with thiol-terminated PEG in AuroVist, enabling contrast loading of 200 mg/mL^29–31^.

As iodine is the most commonly utilized clinical CT contrast agent, many of the micro-CT vascular agents rely on iodine’s attenuating properties. Apart from gold, which has higher attenuation than iodine across the entire energy spectrum, metal-based agents such as alkaline earth metals^21^ and transition metals^32^, have been reported or commercialized. Another class of metals that is ideally suited for micro-CT are the lanthanides (e.g. gadolinium, erbium)^33^, which have k edges (38–64 keV)^34^ that coincide with the peaks of the x-ray energy distributions typically used in high-resolution in vivo micro-CT scanners (typically operating at below 100 kVp). The ideal position of the k-edges offers potential to increase contrast attenuation for single-energy micro-CT scans^33^ and, more importantly, offers the opportunity to match the available x-ray spectrum and contrast agent in dual-energy micro-CT, which is used to distinguish contrast-enhanced vessels from inherently high-attenuating tissues (e.g. bone, calcifications)^35^.

Lanthanide agents designed for in vivo vascular micro-CT are not commercially available, but studies are emerging demonstrating the utility of PEG-modified lanthanide nanoparticles in microimaging^36–39^. To prepare lanthanides that can be used in the in vivo milieu in small animals, common methods include the use of clinically-available MRI agents to formulate nanoparticles that evade rapid renal clearance, or the synthesis of hydrophobic lanthanide nanoparticles encapsulated within a shell of phospholipid-polymer conjugate molecules (*i.e.* liposomes or lipid nanoparticles). While suspending high concentrations of lanthanides (*i.e.* at least 100 mg/mL) in an aqueous environments is challenging, we have recently reported the successful synthesis of block copolymer assembly encapsulations of erbium nanoparticles containing 100 mg/mL of erbium, and demonstrated its utility as a vascular contrast agent when operating at 90 kVp^40^. However, accumulation of the agent within the RES organs resulted in limited viability two days following in vivo contrast agent injection.

The purpose of this work was to synthesize a blood pool contrast agent with at least 100 mg/mL of lanthanide to be cleared by the RES. It is important to note that the extravascular gadolinium-based contrast agents that are used clinically (mainly in MRI) leave the blood pool within minutes of injection and circulate within the extracellular space, while the nanoparticle-based agent that this study developed was intentionally designed to remain in the vasculature over long periods of time and be cleared before the potential release of free gadolinium ions. Instead of encapsulating multiple nanoparticles in block copolymer assemblies that were greater than 100 nm in diameter^40^, we hypothesized that an alternative approach of directly modifying the surface of gadolinium nanoparticles (GdNP) with PEG, would result in smaller polymer-coated nanoparticles, thereby assisting GdNP clearance from RES organs. (Note that gadolinium was selected as the lanthanide to evaluate because it is currently the only lanthanide approved for clinical use, in any form). We demonstrate that such agents can be synthesized, lyophilized and then redispersed to achieve 100 mg/mL of lanthanide in an injectable formulation. Our in vivo results show that the agents provide contrast enhancement values of at least 200 HU in the blood pool for 30 min before being processed in the liver and spleen. The agents were well tolerated, with some gadolinium retention in clearance organs observed 5 days after a high dose injection. To our knowledge, this is the first report on the successful synthesis of colloidally stable aqueous suspensions of gadolinium-based nanoparticles at 100 mg/mL that exhibit sufficient circulation times rendering them suitable contrast agents for vascular imaging by micro-CT.

## Methods

### Reagents and GdNP synthesis details

Details regarding the reagents and their commercial suppliers are available in the Supporting Information (SI). Further details on synthesis and characterization methods are also presented in the SI.

### Contrast agent preparation and physical characterization

#### Synthesis of GdNP

**Figure 1:**
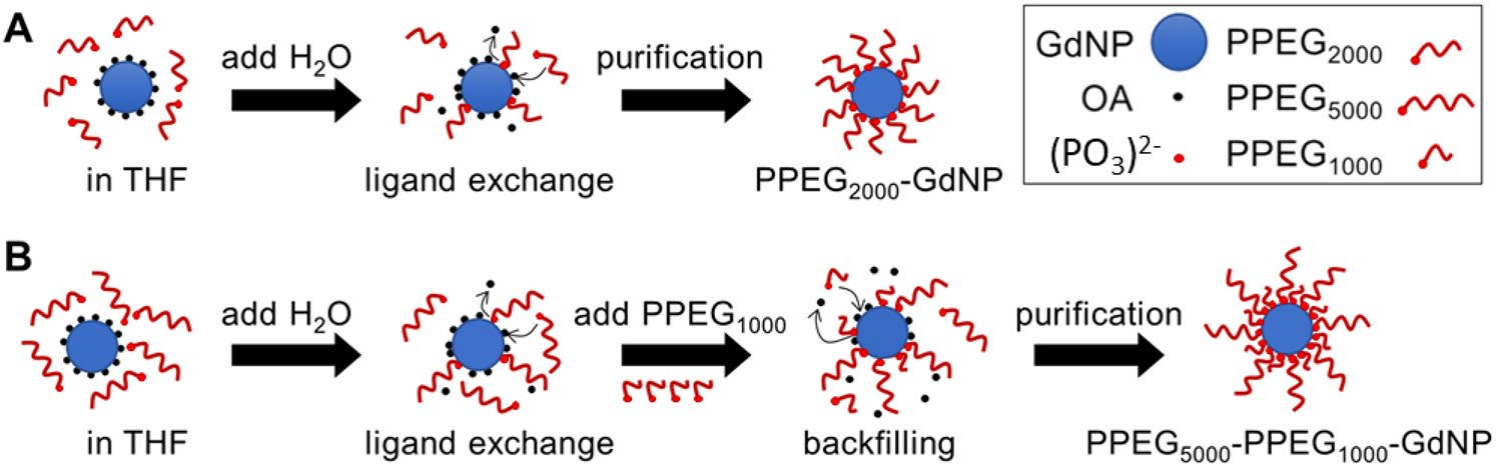
The synthesis of (**A**) PPEG_2000_-GdNP and (**B**) PPEG_5000_-PPEG_1000_-GdNP. OA is displaced by PPEG through ligand exchange, and the solution is purified by dialysis and vacuum sterile filtration to eliminate OA and excess PPEG.

Oleate (OA)-coated NaGdF_4_ (OA-GdNP) were synthesized by a previously reported method^40^. Phosphate-terminated PEG_1000_ (PPEG_1000_), PEG_2000_ (PPEG_2000_), and PEG_5000_ (PPEG_5000_), where the subscripts indicate PEG molar mass in g/mol, were also synthesized as previously reported^41^. Two versions of PPEG-coated GdNP were synthesized—one with PPEG_2000_ only (PPEG_2000_-GdNP), and a formulation using both of PPEG_5000_ and PPEG_1000_ (PPEG_5000_-PPEG_1000_-GdNP). An overview of the PPEG-coating process is presented in Fig. 1. Specifically, GdNP (1.0 g) and PPEG_2000_ (1.0 g) were each dissolved in 12.5 mL of tetrahydrofuran (THF), then combined under magnetic stirring. Deionized water (225 mL) was then added. After stirring for one hour, THF was evaporated and the nanoparticles were purified by dialysis and sterile vacuum filtration (see SI for details). The purified GdNPs were lyophilized and stored at room temperature until they were redispersed immediately prior to use. The same procedure was followed for PPEG_5000_-PPEG_1000_-GdNP, except PPEG_1000_ (1.0 g) dissolved in THF (12.5 mL) was added and the suspension was stirred for one hour before organic solvent evaporation^42^.

#### Physical characterization

The hydrodynamic diameters of the GdNPs were measured by dynamic light scattering (DLS) and were visualized by transmission electron microscopy (TEM). The gadolinium concentration in the contrast agent was measured by inductively coupled plasma-mass spectrometry (ICP-MS) and the results were used to determine the mass of dried contrast agent required to achieve gadolinium concentrations of 100 mg/mL. Micro-CT imaging, where image intensity varies linearly with concentration, was also used to verify the contrast agent concentration (details in SI).

#### Colloidal stability

Saline, which is isotonic with blood, was selected as the solvent for the PPEG-GdNPs. The mouse serum mimic was composed of pH 7.4 phosphate buffered saline, 0.5 µg/mL mouse immunoglobulins, 10 mg/mL bovine serum albumin and 1 mg/mL sodium azide. Colloidal stability in a mouse serum mimic in vitro served as an indicator of stability in the blood in vivo*.* DLS size measurements were performed on the PPEG-GdNP redispersed at 4 mg/mL and the average sizes were observed for up to one hour.

#### Gadolinium release study

To explore the possibility of gadolinium release, PPEG_2000_-GdNP was dialyzed against 1 mM calcium chloride and 0.01 mM zinc chloride in normal saline as dialysate. The gadolinium concentrations in the dialysate were quantified by ICP-MS 1 h, 3 days, and 5 days after the start of dialysis. Measured concentrations were compared to the gadolinium content of the injected agent.

#### Micro-CT scanning and image analysis

Scans were performed using a GE Locus Ultra micro-CT scanner (GE Healthcare, London ON). Whole-body mouse images were acquired over 1000 views (360°, 16 ms per view) at 80 kVp, 55 mA and reconstructed with an isotropic voxel spacing of 150 µm. Images were analyzed using MicroView (Parallax Innovations, London, ON) and CT attenuation was reported in Hounsfield Units (HU). All HU values were measured over a volume of 0.09 mm^3^. The averaged CT attenuation in the bladder (pre-injection) was subtracted from measured tissue attenuations, to report tissue contrast enhancement throughout this paper.

### Contrast agent effect on cell viability

An in vitro cell viability assay was performed using C2C12 mouse myoblast cells, which were incubated for 24 h with contrast agent at concentrations from 0.063 to 1.0 mg/ml. Following incubation, cell viability was measured using a 3-(4,5-dimethylthiazol-2-yl)-2,5-diphenyltetrazolium bromide (MTT) assay. Additional details are provided in the SI.

### In vivo* characterization*

#### Animal care and handling

All animal studies were carried out in accordance with the regulations set out by the Canadian Council on Animal Care, as per an Animal Use Protocol 2018-001 approved by the University of Western Ontario’s Council on Animal Care. C57BL/6 male mice (25–32 g) were purchased from The Jackson Laboratory (Bar Harbor, ME), and were housed in Type II open polycarbonate cage. The animals were kept in temperature and humidity-controlled rooms on a 12 h light–dark cycle, with regular chow and water available ad libitum.

The animals were anesthetized prior to contrast-agent injection and during micro-CT scans using 3.5% isoflurane (Forane, Baxter Corporation, Mississauga, ON) in O_2_ via a nose cone, which was reduced to 1.5% for maintenance.

#### Subcutaneous tissue reaction test

The in vivo toxicity of the contrast agent was evaluated by injecting PPEG_2000_-GdNP and PPEG_5000_-PPEG_1000_-GdNP (0.2 mL, *n* = 2 each formulation) subcutaneously into the dorsal interscapular tissue. The animals were scanned immediately after and two weeks post-injection. The animals were euthanized and dissected for gross tissue observations. Additional details are provided in the SI.

#### Time-course contrast enhancement and biodistribution

The tail veins of seven mice were catheterized using PE-20 polyethylene tubing. PPEG_2000_-GdNP was injected over a period of 3 min (0.2 mL, *n* = 4) while PPEG_5000_-PPEG_1000_-GdNP (0.2 mL, *n* = 3) was injected over a period of 5 min. Micro-CT scans of the animals were obtained at 5, 10, 15, 30, and 60 min post-injection, as well as 2 and 5 days after. The animals were weighed daily during the experiments, until the subjects were euthanized on day 5. Major organs were processed for gadolinium content measurements by ICP-MS and standard histological analyses^43^.

#### Statistical analysis

All values are reported as means ± standard deviations. A two-way repeated-measures analysis of variance (ANOVA) was performed in Prism 8 (GraphPad Software Inc., San Diego, CA, USA) for each formulation to evaluate differences between the contrast enhancement in the vasculature, variations between ICP-MS-measured gadolinium content of excised tissues on day 5, and to measure the effect of intravenous injections to mouse weight. Results were considered statistically significant at *p* < 0.05.

## Results

### Physical characterization

**Figure 2:**
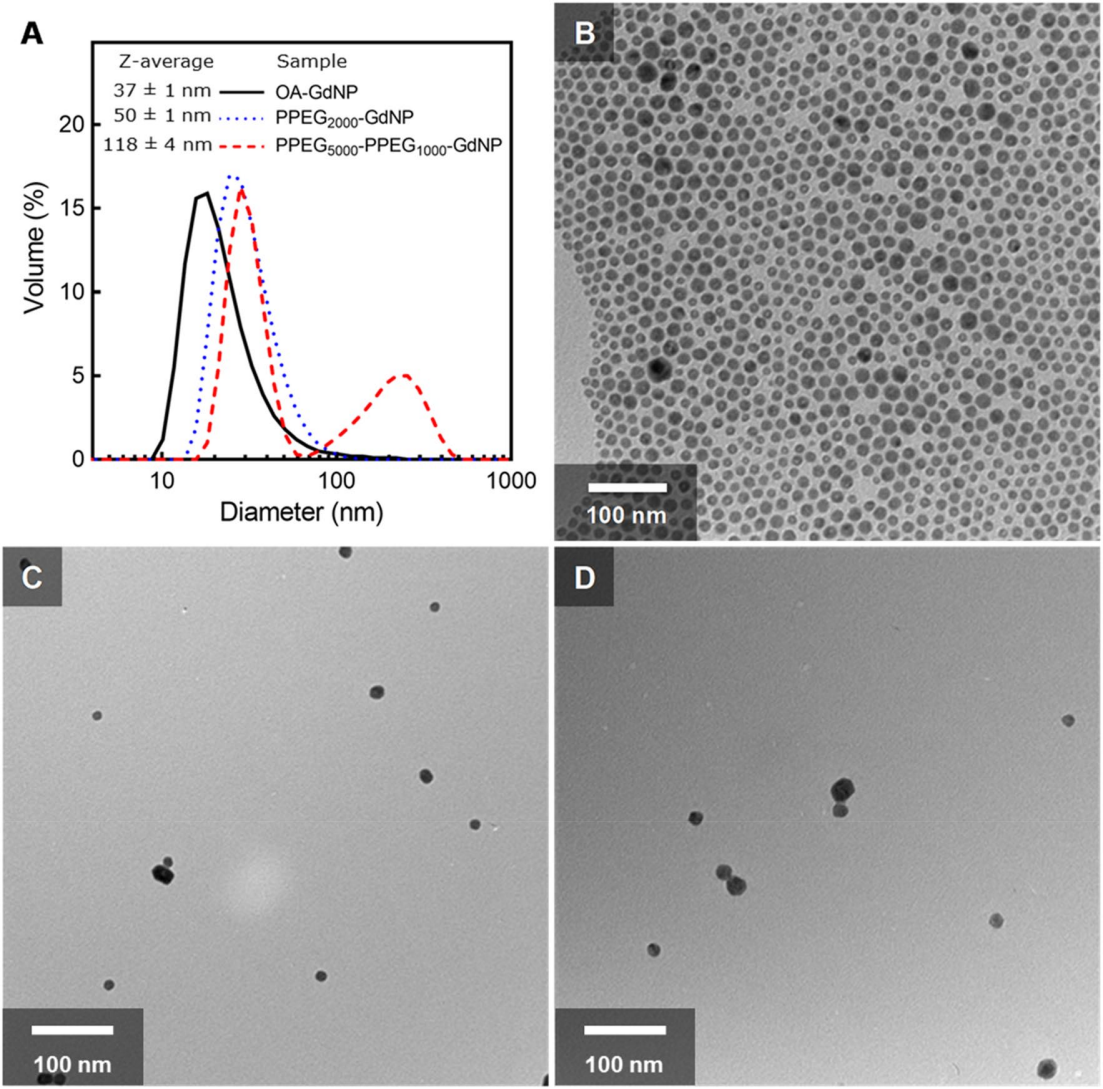
Size distributions and microscopy images of the synthesized GdNPs. (**A**) DLS volume diameter distributions of OA-GdNP in cyclohexane, and PEG_2000_-GdNP and PEG_5000_-PEG_1000_-GdNP in water. Representative TEM images of (**B**) OA-GdNP, (**C**) PEG_2000_-GdNP and (**D**) PEG_5000_-PEG_1000_-GdNP. The scale bar in (**B**) applies to all TEM images.

**Figure 3:**
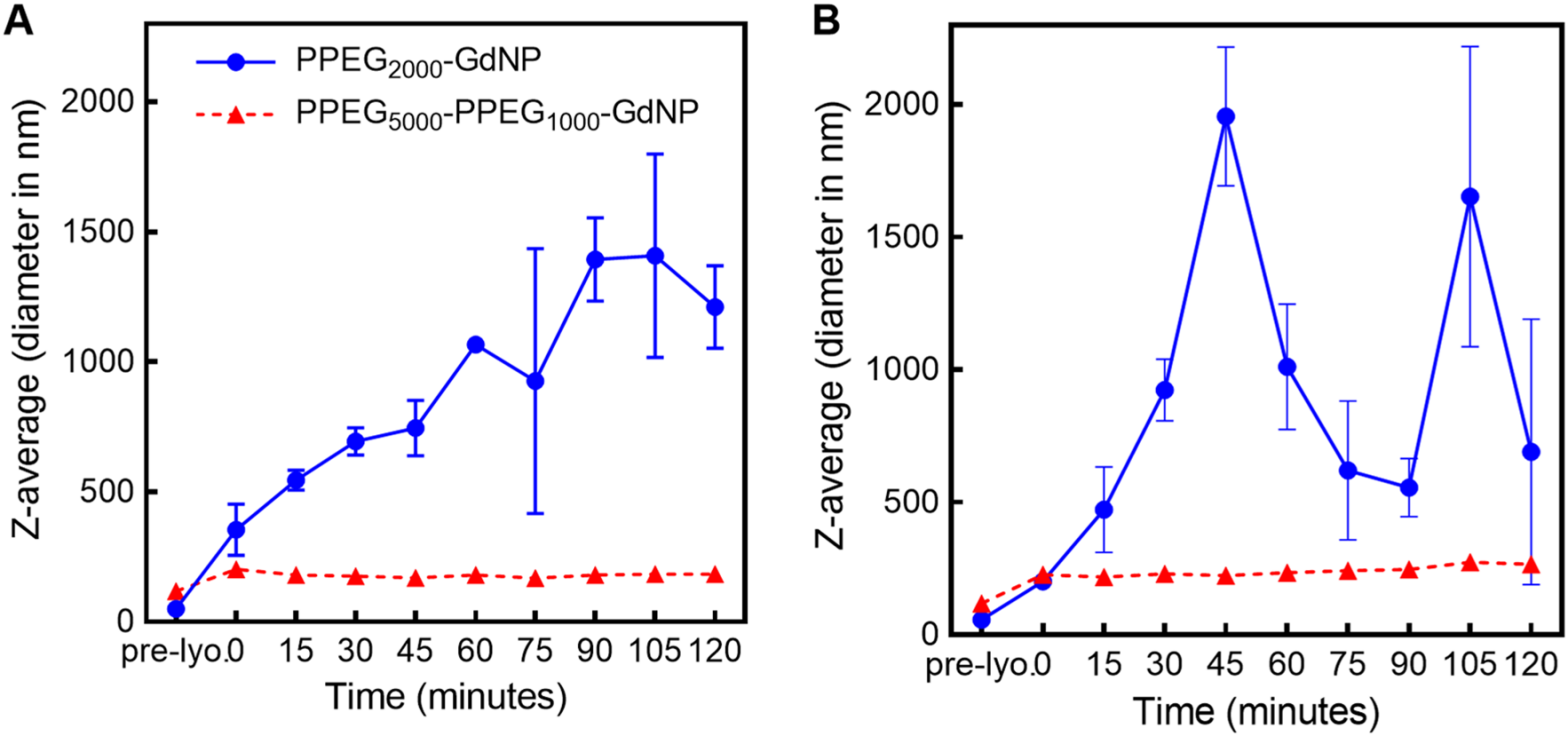
Time-course diameters of resuspended PPEG_2000_-GdNP and PPEG_5000_-PPEG_1000_-GdNP at 37 °C. The contrast agents were redispersed in (**A**) saline and (**B**) a mouse serum mimic for characterization.

OA-GdNP and PPEG_2000_-GdNP had monomodal size distributions, with Z-average diameters of 37 ± 1 nm and 50 ± 1 nm, respectively, both with polydispersity indices (PDI) of 0.20 ± 0.01 (Fig. 2A). PPEG_5000_-PPEG_1000_-GdNP had a bimodal size distribution, with a Z-average diameter of 118 ± 4 nm and a PDI of 0.30 ± 0.01. The particle diameters measured by DLS were in good agreement with those observed in the TEM images (Fig. 2B–D). After lyophilization and redispersion, PPEG_5000_-PPEG_1000_-GdNP had diameters of 202 ± 8 nm in saline and 226 ± 10 nm in a mouse serum mimic (Fig. 3). Colloidal stability was retained for at least 2 h with no substantial changes in Z-average diameters. Larger diameters were observed upon redispersion for PPEG_2000_-GdNP (from 50 ± 1 to 354 ± 99 nm in saline and 201 ± 27 nm in a mouse serum mimic) and the diameters increased over time in saline and mouse serum mimic, as expected.

In the gadolinium release experiment where PPEG_2000_-GdNP was dialyzed against zinc chloride and calcium chloride in saline at biologically relevant concentrations, some gadolinium release was observed. At the 1-h time point, 0.4% of the encapsulated gadolinium was detected in the dialysate. This increased to 3.8% and 7.1% after 3 and 5 days, respectively.

ICP-MS analysis of the contrast agents indicated a gadolinium content of 30 ± 5% and 22 ± 3% (*w/w*) for PPEG_2000_-GdNP and PPEG_5000_-PPEG_1000_-GdNP, respectively. A PEG grafting density of about 4 PEG chains per nm^2^ was calculated for PPEG_2000_-GdNP (supplementary information), while it was not possible to determine the grafting density for PPEG_5000_-PPEG_1000_-GdNP due to the mixture of chain lengths. Hence, to achieve a gadolinium loading of 100 mg/mL in the contrast agent formulation, 66 ± 10 mg of dried PPEG_2000_-GdNP or 90 ± 14 mg of PPEG_5000_-PPEG_1000_-GdNP need to be diluted in 200 µL saline. The relation between CT contrast (HU) and gadolinium concentration in mg/mL ([Gd]) was calculated to be HU = 31[Gd] – 3 (Supplementary Fig. S1). Hence, a contrast value of 3100 HU is anticipated with 100 mg/mL of gadolinium. The CT contrast values obtained from PPEG_2000_-GdNP and PPEG_5000_-PPEG_1000_-GdNP were 3244 ± 57 HU and 3195 ± 62 HU, respectively, corresponding to 105 ± 2 mg/mL and 103 ± 2 mg/mL of gadolinium respectively.

### In vitro and in vivo characterization

In cell culture experiments with PPEG_2000_-GdNP and PPEG_5000_-PPEG_1000_-GdNP, greater than 75% viability of C2C12 mouse myoblast cells was found for all gadolinium concentrations evaluated, up to 1.0 mg/mL (Supplementary Fig. S3); the GdNP are not considered toxic according to the American Society for Testing and Materials^44^. In addition, micro-CT images of the dorsal interscapular region following subcutaneous injection (Supplementary Fig. S4) showed contrast enhancement near the injection site was nearly gone after two weeks. Full body gross examinations showed normal tissues.

**Figure 4:**
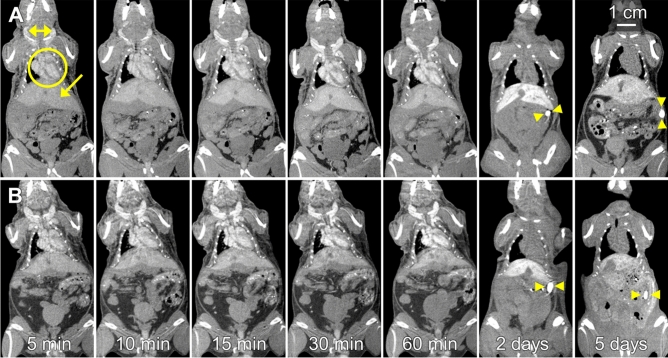
Representative coronal micro-CT images showing the heart, liver, jugular veins, and spleen of mice that received contrast agent formulated with (**A**) PPEG_2000_-GdNP and (**B**) PPEG_5000_-PPEG_1000_-GdNP. All times were reported from the completion of the contrast agent injection. In the 5-min image in (**A**), the blood in the chambers of the heart (circle), liver (arrow) and the external jugular veins (double arrowheads) are clearly visible. In the 2- and 5-day images, the blood pool in the heart is no longer visible, the liver remains visible, and the spleen (arrowheads) becomes visible. Adjacent anatomical slices were shown for the images acquired within 60 min, while slices located 1 cm posterior were shown in the 2- and 5-day images, to demonstrate the high contrast in the previously indistinguishable spleen.

**Figure 5:**
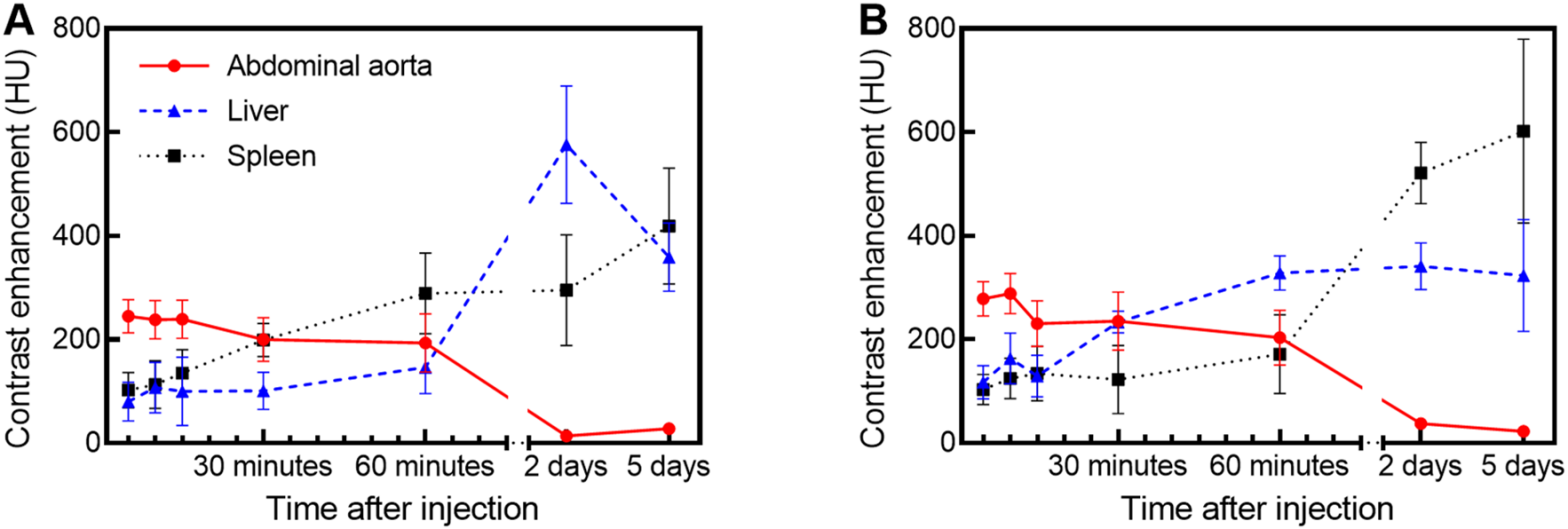
Contrast enhancement in the organs of mice that were injected with (**A**) PPEG_2000_-GdNP and (**B**) PPEG_5000_-PPEG_1000_-GdNP displayed similar trends over time, where decreased attenuation in the vasculature was accompanied by increased attenuation in the liver and the spleen.

Representative time-course micro-CT images of mice injected with the GdNPs are shown in Fig. 4. As expected, qualitative evaluation of the post-contrast images shows opacification of the blood pool (vessels, chambers of the heart), which remained high over the 60 min studied. Increased contrast was observed in the liver as early as 5-min, as demonstrated by the ability to distinguish the liver from surrounding soft tissues; contrast enhancement increased by the 2 day time point. Liver contrast decreased 5 days after agent injection, accompanied by increased contrast in the spleen. The renal cortex was not distinguishable from surrounding tissues, confirming the evasion of renal clearance and clearance via the RES. Quantitative evaluation of the contrast enhancement in the vasculature and the RES organs is shown in Fig. 5. For PPEG_2000_-GdNP, an average attenuation of 245 ± 32 HU was observed 5 min post-injection in the abdominal aorta, while an attenuation of 278 ± 33 HU was observed for PPEG_5000_-PPEG_1000_-GdNP. Values of 200 HU were observed in the blood pool for at least 30 min for both formulations. No significant difference between the measured contrast enhancement of the vasculature for up to 60 min, and no effect on attenuation due to PEG chain length were observed (2-way ANOVA, *p* = 0.15 and 0.077, respectively). Following a single-phase decay model, half-lives were calculated to be 194 and 135 min for PPEG_2000_-GdNP and PPEG_5000_-PPEG_1000_-GdNP, respectively (Supplementary Fig. S2).

**Figure 6:**
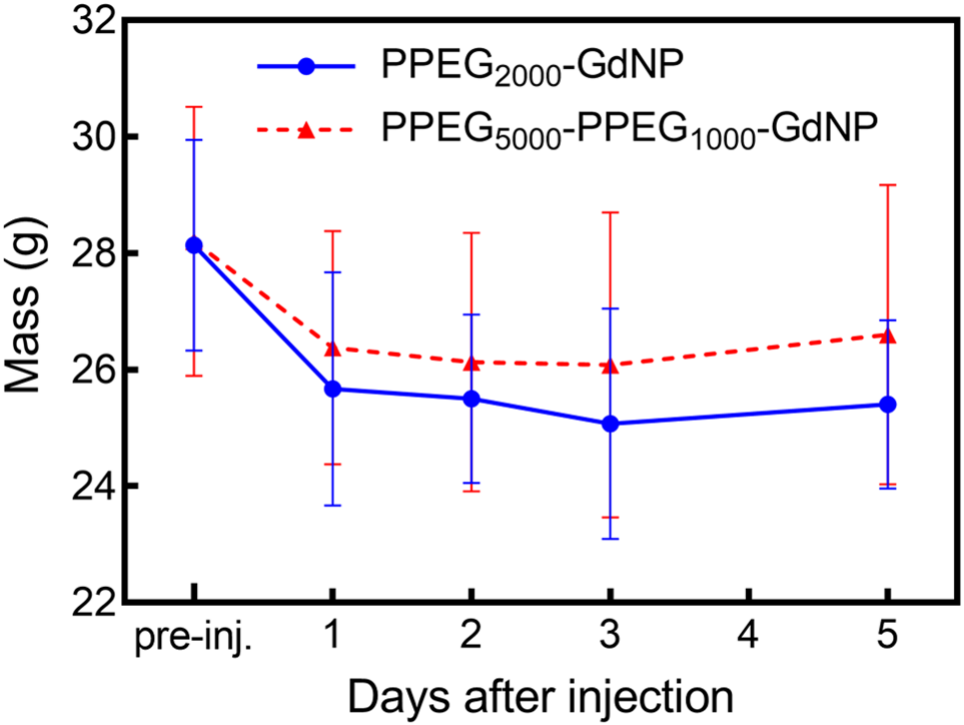
Effect of the contrast-agent injection procedure on subject mass.

The mice were lethargic one day after injection; normal behavior resumed on the second day. The average mass of the injected animals decreased one day after agent injection for both formulations and stopped decreasing after day 3 (Fig. 6). There were no significant differences between daily mass measurements (*p* = 0.30).

Based on post-mortem ICP-MS analyses of the tissues, low gadolinium concentrations were found in the blood, while high gadolinium concentrations were observed in the liver and spleen, as expected (Table 1). The livers and spleens excised from PPEG_5000_-PPEG_1000_-GdNP-injected mice were visibly larger than the organs of mice that were injected with PPEG_2000_-GdNP. While contrast values do not depend on the size or mass of the organ, the total gadolinium contents of the organs were greater for PPEG_5000_-PPEG_1000_-GdNP than PPEG_2000_-GdNP due to organ mass differences (96 ± 22 µg vs. 58 ± 14 µg for the liver, and 68 ± 19 µg vs. 46 ± 11 µg for the spleen, respectively). No gadolinium was found in the heart, kidneys or bladder, but trace gadolinium was measured in the lungs of mice injected with PPEG_5000_-PPEG_1000_-GdNP.

**Table 1:** Gadolinium content of mouse blood and clearance organs determined by ICP-MS.

Tissue	PPEG_2000_-GdNP		PPEG_5000_-PPEG_1000_-GdNP	
	ppm	w/w % of injected gadolinium	ppm	w/w % of injected gadolinium
Blood^a^	1 ± 31	0.1 ± 0.2	5 ± 88	0.1 ± 0.8
Lungs	0	0	52 ± 92	0.1 ± 0.1
Brain	0	0	0	0
Liver	9322 ± 2060	2.7 ± 0.6	10,780 ± 3350	5.2 ± 1.6
Spleen	12,101 ± 2570	2.8 ± 0.6	20,833 ± 2420	7.0 ± 0.8

^a^Reported w/w% were rounded up.

The calibration between CT contrast and gadolinium concentration (Supplementary Fig. S1) confirmed the agreement between HU values (Fig. 5) and the ICP-MS results. The HU values in the liver were calculated to be 298 ± 66 HU and 345 ± 108 HU for PPEG_2000_-GdNP and PPEG_5000_-PPEG_1000_-GdNP, respectively, and were 387 ± 83 HU and 669 ± 78 HU in the spleen. The trace amount of gadolinium found in the lungs by ICP-MS were far lower than the noise, and too low to be detected from the CT images. Significant differences in the gadolinium content values between formulations were observed for the liver (*p* = 0.0006) and the spleen (*p* < 0.0001). Of the injected gadolinium, a total of 5.6 ± 1.0% and 12.4 ± 2.1% remained in the evaluated organs for PPEG_2000_-GdNP and PPEG_5000_-PPEG_1000_-GdNP, respectively.

**Figure 7:**
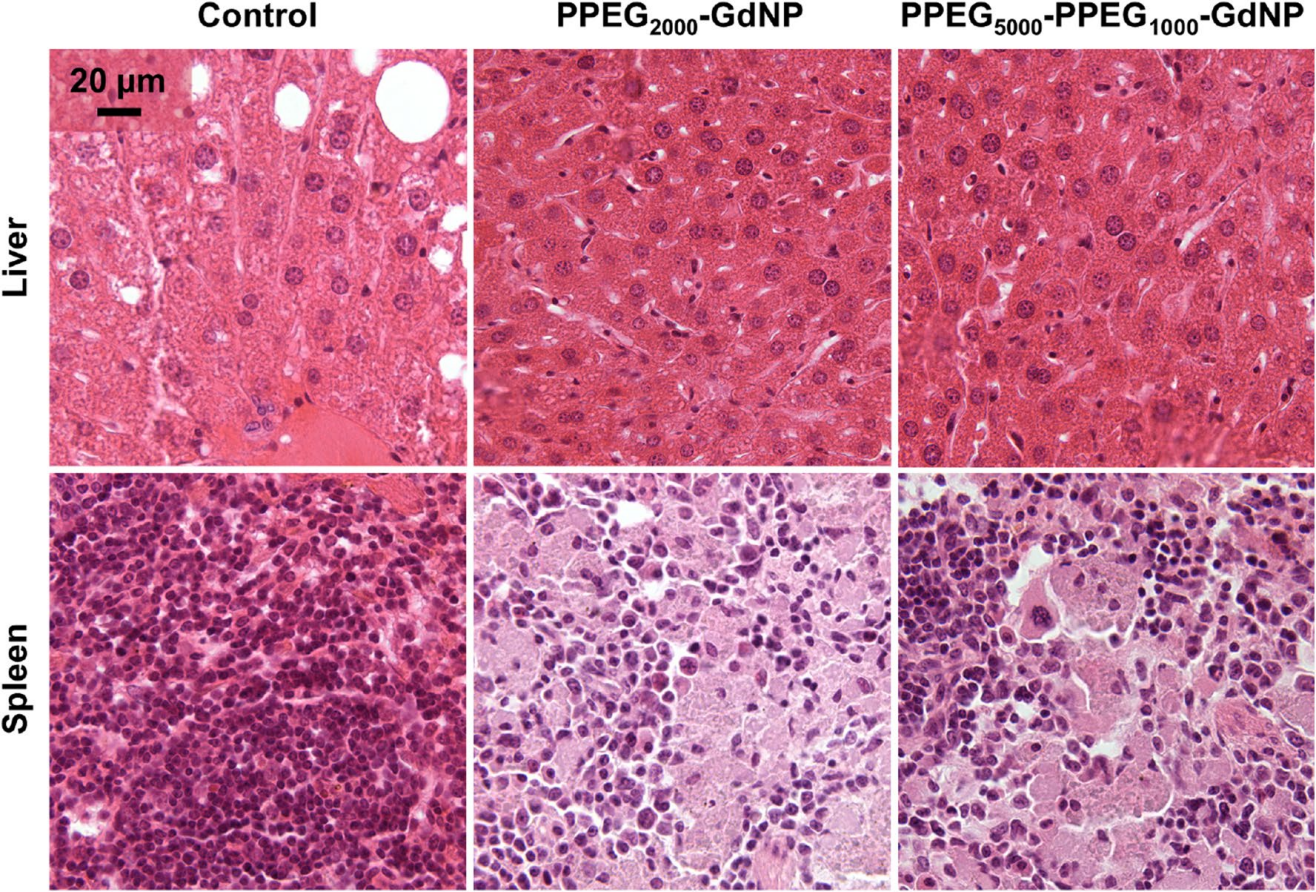
Representative histology images for control and injected mice taken from tissues after 5 days of injection. The scale bar in the liver control section applies to all images. Liver tissues appear normal, while the spleen tissues of the injected mice indicate basophilic nuclear contents in the cytoplasm and lower nuclear densities than the control tissue.

Histological analysis revealed no differences between control and injected mouse liver (Fig. 7), lungs, heart, kidneys and bladder (Supplementary Fig. S5). In spleen tissues, sections from the injected mice demonstrate the presence of basophilic nuclear contents in the cytoplasm and a lower density of nuclei than observed in the control spleen section.

## Discussion

We successfully prepared a long-circulating gadolinium nanoparticle-based vascular micro-CT contrast agent. The nanoparticles can be freeze-dried and redispersed into a contrast agent suspension that is colloidally stable and contains 100 mg/mL of gadolinium, which is difficult to achieve in an aqueous solution. Whole-body CT images demonstrate sufficient vascular enhancement (> 200 HU) over the time period required for scanning, and along with histology and post mortem ICP-MS, confirm RES clearance of both agents. We report the condition of the RES organs days after a high-dose intravenous injection of lanthanide nanoparticles, which has scarcely been reported for other agents. Our results are consistent with an extensive study that demonstrated the performance and in vivo fate of alkaline-earth metal and iodinated nanoparticles when used as vascular contrast agents for micro-CT^45^. Our agent provides comparable vascular contrast and similar clearance pathways as alkaline-earth metal nanoparticles, including their localization in RES organs post-circulation.

Phosphate-terminated PEG was selected to replace the surface oleic acid moieties and render the OA-GdNPs dispersible in water. The phosphate group has higher affinity for Gd^3+^ compared to the carboxylic acid of oleic acid, and the binding of PPEG to the surface of analogous NaYF_4_ nanoparticles has been previously demonstrated using ^31^P NMR spectroscopy^41^. We selected PPEG_2000_ and PPEG_5000_ for this study since these are two of the most commonly used PEG chain lengths in long circulating nanoparticles^46,47^. The grafting density of the PPEG_2000_-GdNP was similar to that observed for gold nanoparticles coated with PEG thiols^48^. While the original goal was to compare the performance of PPEG_2000_-GdNP and PPEG_5000_-GdNP, our preliminary studies showed that PPEG_5000_-GdNP could not be redispersed adequately in aqueous solution. It was suspected that exchange of PPEG_5000_ chains onto the GdNP was more difficult than for PPEG_2000_, thus leaving some of the hydrophobic OA-coated GdNP surface exposed. In attempts to improve the coverage of the GdNP surface, excess PPEG_5000_ of up to five times the mass of the GdNP was used when preparing PPEG_5000_-GdNP. However, the nanoparticles could still not be entirely redispersed in water. Backfilling with a shorter PEG chain length was then adopted, which previous studies have demonstrated to reduce interactions between nanoparticles and plasma proteins^42^. To backfill, PPEG_1000_ was selected as this PEG length was not expected to interfere with the performance of PPEG_5000_ in repelling plasma proteins. The lyophilized PPEG_5000_-PPEG_1000_-GdNP redispersed without difficulty in saline and the mouse serum mimic to form particles with average diameters that were no more than twice the pre-lyophilization diameters. PPEG_2000_-GdNP redispersed at about sevenfold higher diameter than pre-lyophilization. It should be noted that similar PPEG_2000_ coated NaYF_4_ particles were found to be stable with respect to aggregation for weeks to months when incubated in water or cell culture media. However, the lyophilization process that we used, which is needed to obtain the high concentrations required for CT, may hinder our ability to completely redisperse the dry materials into individual particles in solution, and may expose hydrophobic sites that lead to gradual aggregation^41^.

The PPEG_5000_-PPEG_1000_-GdNP formulation was observed (qualitatively) to have a higher viscosity than PPEG_2000_-GdNP at concentrations of 100 mg/mL of gadolinium. This resulted in longer injection times for PPEG_5000_-PPEG_1000_-GdNP, requiring 5-min injections versus the 3-min injections when using PPEG_2000_-GdNP. The PPEG_5000_-PPEG_1000_-GdNP had a lower gadolinium content (22 ± 3% w/w) compared to the PPEG_2000_-GdNP (30 ± 5%). Therefore, more PPEG_5000_-PPEG_1000_-GdNP on a mass/volume ratio was required to achieve 100 mg/mL of gadolinium for the injection. Since the viscosity of a suspension containing solid spherical particles increases when a larger volume of the solution is occupied by the particles/polymer, the viscosity was higher for PPEG_5000_-PPEG_1000_-GdNP^49^. The longer PPEG_5000_ chains on the particle surface may also contribute to higher viscosity compared to PPEG_2000_.

Similar contrast enhancement of the vasculature (above 200 HU) was achieved for 30 min after injection for both formulations, with no significant change in time-course attenuation for up to 60 min. The similarity in in vivo performance of the reported formulations was initially unexpected based on the in vitro studies in saline and the mouse serum mimic because PPEG_2000_-GdNP showed poorer colloidal stability than PPEG_5000_-PPEG_1000_-GdNP. However, the in vivo behavior of nanoparticles is largely determined by the protein corona, a complex multilayered structure, with a composition that is determined by the initial nanoparticle surface chemistry as well as factors such as sheer stresses experienced in the blood and extravasation within blood vessels^50,51^. Protein coronas differ between in vitro and in vivo studies so in vitro tests cannot fully predict the in vivo behavior of nanoparticles in the vasculature. The nanoparticles reported in this paper exhibited the same circulation times and blood pool contrast enhancements as our previous formulation comprising NaErF_4_ nanoparticles encapsulated in assemblies formed from PEG-poly(lactic acid) block copolymers^40^, which also had PEG surfaces. This current study and our previous paper are further testaments to the capabilities of PEG in temporarily promoting stealth properties against the RES and in providing its cargo with long circulation times in the vasculature, even at the high doses required for micro-CT.

No CT scans were performed after the first 60 min on day one or the day after injections to avoid complicating mouse health due to anesthesia^52^. The injected mice were clearly lethargic after being under anesthesia for 60 min, and this effect was still observed after one day. Two days following initial injection, the mice were placed under anesthesia for 5 min, only to permit micro-CT imaging; the mice were more active before and after imaging, and only needed a few minutes to recover from anesthesia. The daily mass measurements exhibit a similar trend that coincides with mouse activity, food and water intake. While a limitation of this study is that no sham study was performed to differentiate between the effect of the agent and the anesthesia, isoflurane was found to increase mouse latency in previous studies and coincided with decreased animal weight for up to 2 days^53^.

Our results—micro-CT time course and post-mortem ICP-MS of relevant organs—clearly demonstrate that the agent’s clearance pathway is through the RES. This was expected due to its physical properties and is similar to the clearance of other agents incorporating metal nanoparticles^32,36^. Some gadolinium was detected by ICP-MS in trace amounts in the blood, and in the lungs of PPEG_5000_-PPEG_1000_-GdNP-injected mice; the trace values found in the lungs can be attributed to reversible transient aggregation in capillary beds that other intravenously injected nanoparticles also demonstrated^54,55^. Because of concerns with free gadolinium being observed in humans post small-molecule contrast injection^56,57^, we evaluated the gadolinium concentration in the brain and confirmed that our GdNP did not cross the blood brain barrier.

The ICP-MS results reported significantly higher gadolinium accumulation in the liver and in the spleen from PPEG_5000_-PPEG_1000_-GdNP than PPEG_2000_-GdNP. This can be attributed to a difference in PEG properties once grafted onto nanoparticle surfaces, specifically the morphology and grafting density, which was previously reported with gold nanoparticles^58,59^. Considering greater RES organ accumulation, higher viscosity, and heavier excised livers and spleens from PPEG_5000_-PPEG_1000_-GdNP injections than from PPEG_2000_-GdNP, and no significant difference in time-course contrast enhancements of the vasculature, a greater advantage can be gained from using PPEG_2000_-GdNP instead of PPEG_5000_-PPEG_1000_-GdNP for in vivo micro-CT. This was an unexpected result, because PPEG_5000_-PPEG_1000_-GdNP particle suspensions were more stable in vitro. We suspect that the size transience of PPEG_2000_-GdNP—demonstrated within minutes of incubation with saline and a mouse serum mimic—promoted mobility and reduced accumulation in RES organs. This has been previously demonstrated by other researchers that studied nanogels of varying rigidity, reporting greater particle mobility and lower accumulation in clearance organs with less rigid nanogels compared to more rigid nanogels^60,61^.

Optical histology results showed that all tissues examined, except for the spleen, were normal. The gadolinium release experiment demonstrated the possibility of released ions causing toxicity in the spleen, although we did perform the experiments using concentrated salts in the dialysate. Nonetheless, the particles are much more stable with respect to the release of free gadolinium ions than small molecule gadolinium agents.

In this study, mice were euthanized after the 5-day time point because after that time point it is difficult to gauge if well-being is a result of the contrast agent or other factors. The contrast agent formulation that we report in this paper presents an improvement from our previously reported nanoparticle assembly formulation, where the majority of mice died after 2 days. While further studies are required to enable the contrast agent’s utility in long-term studies, our results show that switching from diblock polymer coated nanoparticle assemblies to grafting PEG directly on the surface—preferably with PEG_2000_—facilitated the exit of nanoparticles from the liver, which will ultimately be important to ensure that the particles are cleared from the body before the eventual breakdown of the nanoparticles and the potential release of Gd^3+^ ions.

## Conclusions

We synthesized long-circulating contrast agents composed of PEG-modified GdNP that can be suspended at 100 mg/mL of gadolinium and can be used to visualize the vasculature of live mice using micro-CT for up to 60 min. The alternative approach of modifying the surface of the nanoparticles with PEG in lieu of polymer-coated nanoparticle assemblies presents a step in the right direction towards making micro-CT contrast agents available for long-term longitudinal vascular research.

## Supplementary Information

Supplementary Information.

## Data Availability

Data sets are available upon request from the corresponding author.
