# Supplementary material for: PEG-modified gadolinium nanoparticles as contrast agents for in vivo micro-CT
Source: Sci Rep. 2021 Aug 16;11:16603. doi: 10.1038/s41598-021-95716-x (PMC8367985; doi:10.1038/s41598-021-95716-x)
Supplement: Supplementary file 1 — Supplementary Information. [file 41598_2021_95716_MOESM1_ESM.pdf]

Supporting material for:

PEG-modified gadolinium nanoparticles as contrast agents for *in vivo* micro-CT

Charmainne Cruje,[a,b] P. Joy Dunmore-Buyze,[a] Eric Grolman,[a,c] David W. Holdsworth,[a,b,c] Elizabeth R. Gillies,[c,d,e] and Maria Drangova[a,b,c#]

[a]Robarts Research Institute, [b]Department of Medical Biophysics, [c]School of Biomedical Engineering, [d]Department of Chemistry, [e]Department of Chemical and Biochemical Engineering, Western University, London, Ontario, Canada N6A 5B7

[#]corresponding author

Supplementary materials and methods. General materials – Gadolinium chloride hexahydrate, PEG monomethyl ether (mPEG, M_n = 1000, 2000, 5000), and 450 nm cellulose acetate membrane syringe filters were purchased from Millipore Sigma (Oakville, ON). Sterile disposable filter units with a 450 nm pore size were purchased from ThermoFisher Scientific (Ottawa, ON). Ammonium fluoride, oleic acid, 1-octadecene, phosphoryl trichloride, calcium chloride, and zinc chloride were purchased from Alfa Aesar (Ward Hill, MA). Sodium hydroxide, hexane, tetrahydrofuran (THF), hydrochloric acid, and nitric acid were purchased from Caledon Laboratories (Georgetown, ON). Ethanol was purchased from Commercial Alcohols (Tiverton, ON). Dry toluene was obtained from a solvent purification system and was stored over molecular sieves. PEG was dried by heating at 110 °C for 1 hour under high vacuum immediately before use. A gadolinium standard with a concentration of 10,000 µg/mL was purchased from Delta Scientific Laboratory Products Ltd. (Mississauga, ON). Spectra/Por 6 dialysis tubing (50 kDa MWCO) was obtained from Spectrum Laboratories (Rancho Dominguez, CA). Normal saline (0.9 % NaCl) was purchased from Cardinal Health (Mississauga, ON). Mouse Primary Antibody Isotype Control, which is a mouse serum mimic, fetal bovine serum (FBS), Glutamax (100X) solution Penstrep (100X), and Dulbecco's modified Eagle's medium were obtained from Invitrogen Corporation (Camarillo, CA). C57BL/6 male mice (25–30 g) were purchased from Jackson Laboratories (Bar Harbor, ME), and isoflurane from Baxter Corporation (Mississauga, ON).

General characterization methods – The nanoparticle hydrodynamic diameters were measured by dynamic light scattering (DLS) using a Zetasizer Nano ZS instrument (Malvern Instruments Ltd, Malvern UK) at room temperature (37°C) in a quartz cuvette (1 mg/mL). Transmission electron microscopy (TEM) was performed using a Philips CM10 (Philips, Amsterdam) using an acceleration voltage of 80 kV. A drop from a suspension of the samples in cyclohexane or water (1 mg/mL) was deposited onto a copper grid and air-dried overnight. Samples were centrifuged in 50 mL tubes using a VWR Clinical 200 centrifuge with a 28° fixed-angle rotor. The gadolinium content of samples that were digested and preserved with aqua regia[1] (2%) were measured by inductively coupled plasma mass spectrometry (ICP-MS) using the Agilent 1260 Infinity HPLC connected directly to a new Agilent 7700 Series ICP-MS (Santa Clara, CA).

Synthesis of gadolinium nanoparticles (GdNP) – Specifically, the GdNP in this study are $NaGdF_4$ nanocrystals and were synthesized based on a previously reported method with some modifications.[2,3] Gadolinium chloride hexahydrate (2.2 g, 6.0 mmol, 1.0 equiv), oleic acid (90 mL), and 1-octadecene (90 mL) were magnetically stirred and subjected to high vacuum. The

resulting mixture was heated to 120°C for 1 hour, then cooled to 50 °C under argon. Ammonium fluoride (900 mg, 24.5 mmol, 4.0 equiv) and sodium hydroxide (600 mg, 15 mmol, 2.5 equiv) were dissolved in 50 mL of methanol and added dropwise to the cooled mixture. The solution was kept under the same conditions for 30 minutes. It was then heated to 65 °C under vacuum for 30 minutes to evaporate methanol, and then to 100 °C for another 30 minutes to evaporate water. High vacuum was removed, and the mixture was placed under an argon atmosphere. The mixture was heated to 300 °C for 2 hours and cooled to room temperature for purification.

To precipitate the nanoparticles, ethanol was added to the resulting solution at a 5:1 volume ratio and centrifuged at 6000 rpm for 30 minutes. The supernatant was decanted, the nanoparticles were redispersed in THF, and the solution was centrifuged again. Sedimented nanoparticles were dried under vacuum overnight and stored at room temperature. A 1 mg/mL sample of the nanoparticles was prepared in cyclohexane for characterization by DLS and TEM.

Purification of PEG-coated nanoparticles – The solution containing PEG-coated GdNP were dialyzed against 4 L of milliQ water over 24 hours, with 5 dialysate changes. The resulting solution was filtered *via* sterile vacuum filtration (450 nm pore size), and then lyophilized.

Calculation of PEG grafting density for $PPEG_{2000}$-GdNP – The calculation was performed as previously reported for the grafting density of thiol-terminated PEG on gold nanoparticles,[4] except that a density of 7 g/cm^3 was used,[5] and the polymer versus nanoparticle content was determined based on the ICP-MS analysis indicating 30% w/w gadolinium. A mean $NaGdF_4$ core particle diameter of 12.7 ± 2.3 nm (based on the measurement of 75 particles by TEM) was used in the calculation.

Linear regression of CT contrast and gadolinium concentration – The relationship between CT contrast and gadolinium concentration was first confirmed by micro-CT. Gadolinium chloride was diluted in saline at gadolinium concentrations of 5, 10, 15 and 100 mg/mL, which acted as calibration standards. The dried contrast agent was dissolved in saline to contain 100 mg/mL of gadolinium; the mass of dried contrast agent required to obtain 100 mg/mL of gadolinium was determined from ICP-MS data. The linear regression between CT contrast (in HU) and gadolinium concentration was then used to calculate the gadolinium content of the contrast agent.

Cell viability assay – C2C12 mouse myoblast cells were seeded in a Nunclon 96-well U-bottom transparent polystyrol plate to obtain approximately 10,000 cells/well in 100 µL of Dulbecco's modified Eagle's medium containing serum, glutamax, and antibiotics. The cells were allowed to adhere to the plate in a 5% CO_2 incubator at 37 °C for 24 h. The growth medium was then aspirated and replaced with either solutions of sodium dodecyl sulfate (SDS) in the cell culture medium at concentrations of 0.2, 0.15, 0.10, or 0.05 mg/mL, which were used as positive controls, serial dilutions of the contrast agent, or fresh medium. The cells were then incubated at 37°C (5% CO2) for 24 h. The medium was again aspirated and replaced with 110 µL of fresh medium containing 0.5 mg/mL (3-(4,5-dimethylthiazol-2-yl)-2,5-diphenyltetrazolium bromide) (MTT). After 4 h of incubation (37°C, 5% CO_2), the MTT solution was carefully aspirated and the purple crystals were dissolved by addition of 50 µL of spectroscopic grade dimethyl sulfoxide (DMSO). After shaking (1 s, 2 mm amp, 654 rpm), the absorbance of the wells at 540 nm was read using an M1000-Pro plate reader (Tecan). The absorbance of wells prepared in the same way but without cells was

subtracted as a background and the cell viability was calculated relative to wells containing cells that were exposed only to the culture medium. Cell viability was detected for cells exposed to only to the lowest concentrations of SDS, confirming the sensitivity of the assay.

***In vivo* reaction test in subcutaneous tissue** – C57BL/6 mice (25-32 g) were injected with 0.2 mL of saline (n=2), $PPEG_{2000}$-GdNP (n=2), or $PPEG_{5000}$-$PPEG_{1000}$-GdNP (n=2) subcutaneously, in the dorsal interscapular region. These animals were scanned immediately after injection, and two weeks after. The animals were scanned using general micro-CT imaging and analysis methods as described in the manuscript. Representative images of a mouse from each group are shown in Fig. S4. While both formulations of the contrast agent localized near the injection site for up to two weeks, lower contrast was visualized at the two-week timepoint, suggesting clearance of the contrast agent over time after injection. The animals were sacrificed at the two-week timepoint for subcutaneous, dermal tissue and clearance organ gross examination. All tissues appeared normal.

Supplementary results

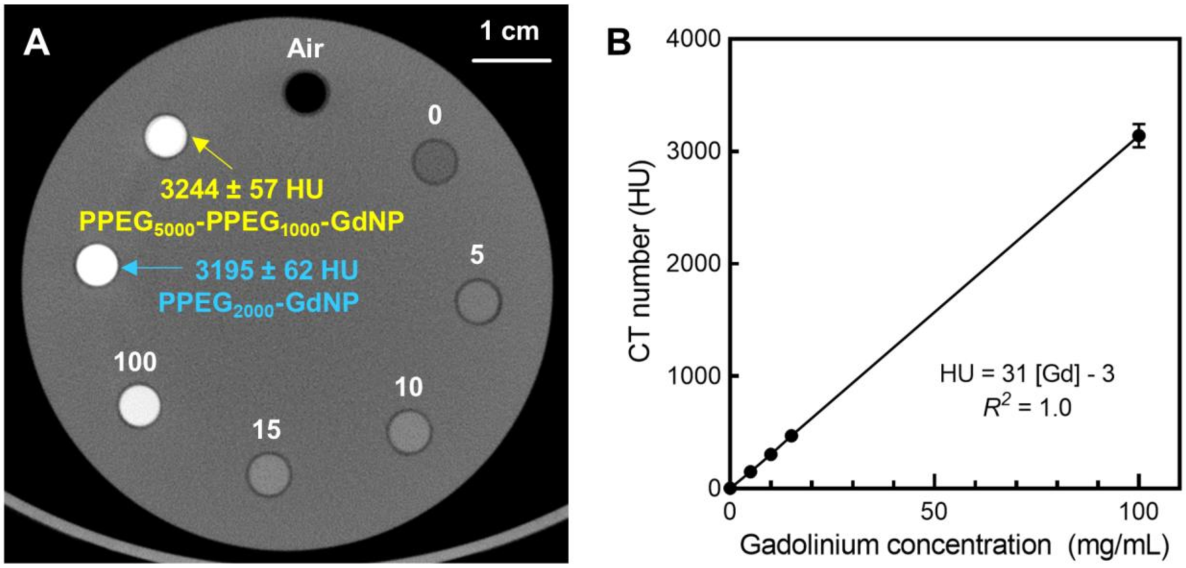

Figure S1. A) Micro-CT image (at 80 kVp) of calibration standards containing known concentrations of gadolinium chloride, which were used to determine the gadolinium content of the $PPEG_{2000}$-GdNP and $PPEG_{5000}$-$PPEG_{1000}$-GdNP. B) Measured CT number vs. known gadolinium concentration. The CT numbers of the concentrated solutions verified the suspension of 100 mg/mL of gadolinium in each of the formulations. C) *In vitro* cell viability as determined by MTT assay. No effect on viability was observed.

Blood pool half-life calculation – The time-course contrast enhancement in the vasculature was modelled using a one-phase decay model (Fig. S2). The decay rate k of each line of best fit were 0.00358 and 0.00513 for $PPEG_{2000}$-GdNP and $PPEG_{5000}$-$PPEG_{1000}$-GdNP, respectively. Half-lives $t_{1/2}$ were calculated as $ln(2)/k$, which yield 194 and 135 minutes, respectively. These calculated half-lives are longer than those of clinical gadolinium-based contrast agents, which are in the order of minutes following a two-phase decay model.[6]

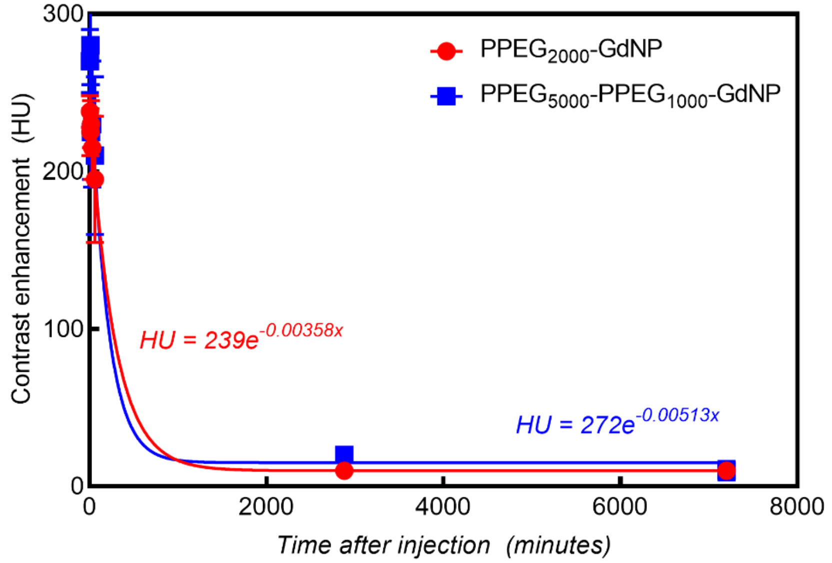

Figure S2. Blood pool contrast enhancement fitted to single-phase exponential decay model. The half-lives of $PPEG_{2000}$-GdNP and $PPEG_{5000}$-$PPEG_{1000}$-GdNP in the blood were calculated to be 194 and 135 minutes, respectively.

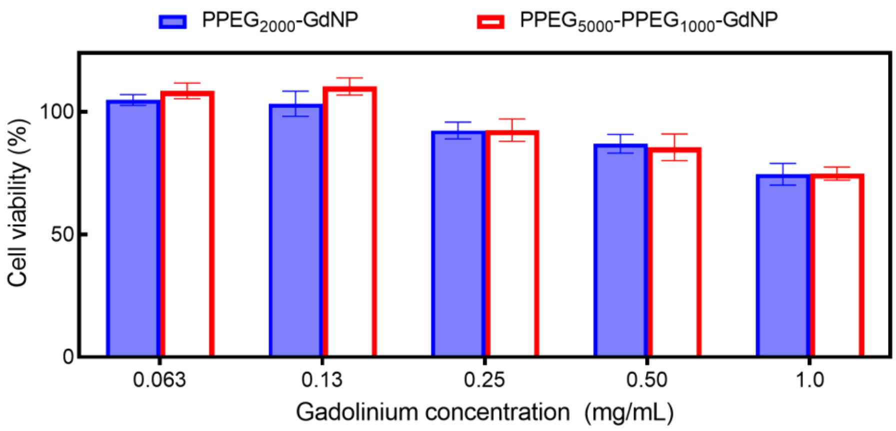

Figure S3. *In vitro* toxicity as determined by the MTT assay of the contrast agent. $PPEG_{2000}$-GdNP and $PPEG_{5000}$-$PPEG_{1000}$-GdNP did not cause toxicities to mouse myoblast cells *in vitro*.

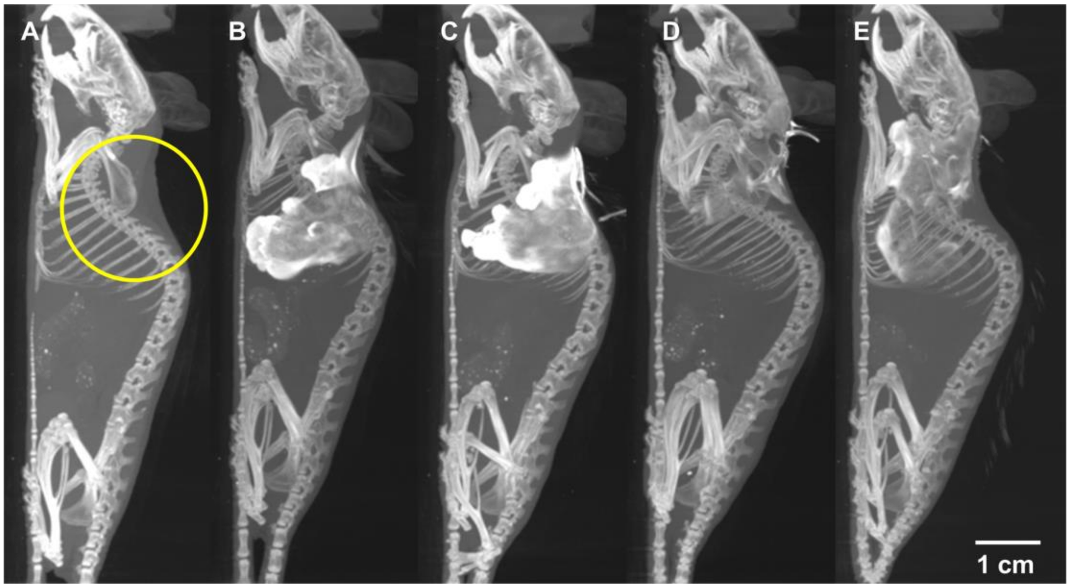

Figure S4. Thick maximum intensity projections (5 cm) of mice injected subcutaneously. Representative subjects were imaged immediately after injection with A) saline, B) $PPEG_{2000}$-GdNP, and C) $PPEG_{5000}$-$PPEG_{1000}$-GdNP. Images were also taken 2 weeks post-injections with D) $PPEG_{2000}$-GdNP, and E) $PPEG_{5000}$-$PPEG_{1000}$-GdNP. The injection site (*i.e.* dorsal interscapular region) is identified by the circle in A.

Histological analysis of excised tissues – Hematoxylin stains the nucleus purple, while eosin counterstains the cytosol and extracellular matrix pink.[7] The heart, kidney and bladder tissues of injected mice demonstrate no difference from control mouse tissues. Despite high gadolinium concentrations in the liver, no difference in histological images were observed. The trace amounts of gadolinium that were found in the lungs of $PPEG_{5000}$-$PPEG_{1000}$-GdNP-injected mice did not result in notable differences. Contrasting histological staining is demonstrated in the spleen, where control tissues have higher nuclear density than $PPEG_{2000}$-GdNP- and $PPEG_{5000}$-$PPEG_{1000}$-GdNP-injected mice. The spleen sections from the control mouse demonstrates a deeper pink stain than the sections from injected mice, which appear purple. Although representative sections are displayed in Fig. S5, similar images were observed from different regions of the excised tissue.

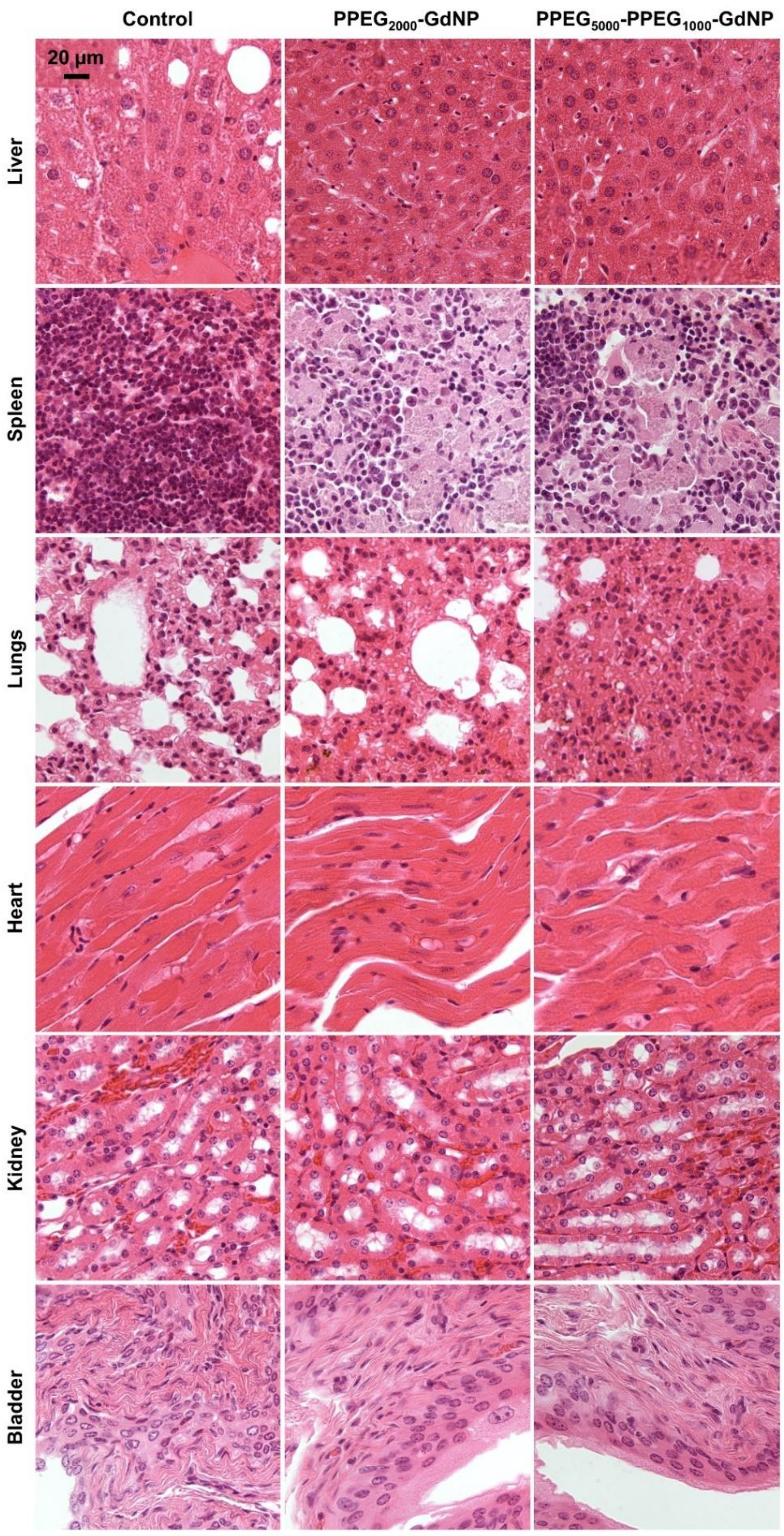

Figure S5. Representative histology images for control and injected mice taken from tissues after 5 days of injection. The liver, lung, heart, kidney, and bladder tissues appear normal, while spleen tissue of the injected mice appear more purple than the staining observed in the control mouse, which appears pink.

References

1 Dufort, S. *et al.* The High Radiosensitizing Efficiency of a Trace of Gadolinium-Based Nanoparticles in Tumors. *Sci Rep* **6**, 29678, doi:10.1038/srep29678 (2016).

2 Li, Z. & Zhang, Y. An efficient and user-friendly method for the synthesis of hexagonal-phase NaYF(4):Yb, Er/Tm nanocrystals with controllable shape and upconversion fluorescence. *Nanotechnology* **19**, 345606-345610, doi:10.1088/0957-4484/19/34/345606 (2008).

3 Zhao, G., Tong, L., Cao, P., Nitz, M. & Winnik, M. A. Functional PEG-PAMAM-tetraphosphonate capped NaLnF(4) nanoparticles and their colloidal stability in phosphate buffer. *Langmuir* **30**, 6980-6989, doi:10.1021/la501142v (2014).

4 Benoit, D. N. *et al.* Measuring the grafting density of nanoparticles in solution by analytical ultracentrifugation and total organic carbon analysis. *Anal Chem* **84**, 9238-9245, doi:10.1021/ac301980a (2012).

5 Weber, M. J. Inorganic scintillators: today and tomorrow. *Journal of Luminescence* **100**, 35-45, doi:https://doi.org/10.1016/S0022-2313(02)00423-4 (2002).

6 Tombach, B. & Heindel, W. Value of 1.0-M gadolinium chelates: review of preclinical and clinical data on gadobutrol. *European Radiology* **12**, 1550-1556, doi:10.1007/s00330-001-1242-9 (2002).

7 *Theory and practice of histological techniques / [edited by] John D. Bancroft, Marilyn Gamble*. 6th ed. edn, (Churchill Livingstone Elsevier, 2008).
